# Synthetic Microbial Surrogates Consisting of Lipid Nanoparticles Encapsulating DNA for the Validation of Surface Disinfection Procedures

**DOI:** 10.1021/acsabm.3c00004

**Published:** 2023-02-28

**Authors:** Lara Pfuderer, Wendelin J. Stark, Robert N. Grass

**Affiliations:** Institute for Chemical and Bioengineering, ETH Zurich, Vladimir-Prelog-Weg 1, 8093 Zurich, Switzerland

**Keywords:** LNP, nanoparticles, tracing, DNA, hygiene

## Abstract

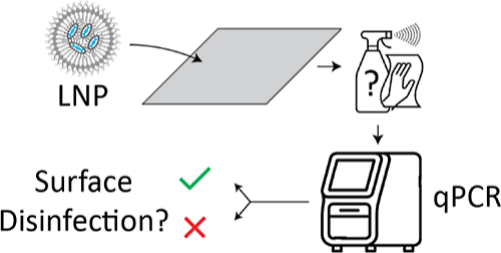

Effective cleaning and disinfection procedures are an integral part of good manufacturing practice and in maintaining hygiene standards in health-care facilities. In this study, a method to validate such cleaning and disinfection procedures of surfaces was established employing lipid nanoparticles (LNPs) encapsulating DNA. It was possible to determine and distinguish between the physical cleaning effect (dilution) and the chemical cleaning effect (disintegration) on the LNPs during the cleaning and disinfection procedure (wiping). After treatment with 70 v % ethanol as a disinfectant and SDS solution as a cleaning agent, LNPs showed log_10_ reductions of 4.5 and 4.0, respectively. These values are similar to the log_10_ reductions exhibited by common bacteria, such as *Escherichia coli* and *Serratia marcescens.* Therefore, LNPs pose as useful tools for cleaning validation with advantages over the already existing tools and enable a separate detection of dilution and chemical disinfectant action.

## Introduction

Cleaning and disinfection of surfaces is an integral part of good manufacturing practice, which is applied in the manufacturing of food, beverages, pharmaceutical products, and medical devices.^1−3^ In health-care settings, the disinfection of surfaces is of great importance as well, as transmission events of pathogens via health-care workers’ hands from surfaces to patients and vice versa are problematic and can lead to health-care-associated infections.^4^ Possible consequences of health-care-associated infections are prolonged hospital stays, additive financial burden, and excess death rates.^5^

The validation of cleaning and disinfection procedures of surfaces is necessary to ensure the desired hygienic standards. The current standard method to determine the general effectiveness of a cleaning procedure is the application of dye markers. To show that a specific surface is free from bacterial or fungal contamination, more advanced tools such as ATP tests and agar contact plates are routinely used. Although these methods are widely used and make up the market for surface cleaning validations, they possess several limitations concerning sensitivity, interferences, and time demands.^6^ As such, dyes can only show if a surface was rinsed, not if the cleaning procedure was intensive enough to remove pathogens.^7^ Similarly, ATP tests and agar contact plates are not able to detect very low pathogen concentrations and are not sensitive to viruses, prions, or slowly dividing bacteria.^8,9^

In this work, we set out to find a suitable alternative method for the validation of cleaning and disinfection of surfaces. It should show a high sensitivity toward disinfection in order to be a direct measure for the cleanliness of surfaces. Ideally, such a tool should not only be able to detect whether a surface was cleaned or wiped, but it should also be able to detect whether an appropriate chemical disinfectant, such as ethanol solutions or surfactants, were applied in the cleaning procedures.

In the formulation of such chemical disinfection products, the selective action of the disinfectant to destroy or inhibit microorganisms is maximized while otherwise being as non-hazardous and cost-effective as possible. Ideal solutions to this are the use of detergents and ethanol- or isopropanol-based disinfecting agents as these lead to the disruption of the lipid membranes of pathogens, thereby performing the anti-microbial effect.^10,11^

A most primitive idea would, therefore, be to directly use microorganisms for the validation of cleaning procedures. However, this implies that either microorganisms must be intentionally deployed, which might lead to unforeseen side effects, or the method has to rely on the general presence of such microorganisms. While this is a prerequisite for ATP-based tests and agar contact plates, cleaning validation procedures are often employed in areas with no or very low contamination levels (e.g., clean rooms or manufacturing equipment).

While benign surrogate microorganisms have been proposed,^12,13^ a non-living surrogate would be ideal. Such a synthetic surrogate should be comparable to a given microorganism in sensitivity to general disinfection products but be essentially harmless. Also, the detection of the synthetic surrogate should be as sensitive as possible, ideally having a detection sensitivity comparable to the detection of common microorganisms (detection of individual infective units, CFU).

When selecting such a synthetic surrogate, we first evaluated the use of liposomes. Their membrane is comparable to the membrane of Gram-negative bacteria and to enveloped viruses. However, due to the extreme sensitivity of liposomes toward desiccation, these ideas were rapidly discarded. Instead, we evaluated the use of lipid nanoparticles (LNPs) as synthetic surrogates as they are also somewhat similar to microorganisms in size range and surface chemistry but are significantly more robust than liposomes. Such LNPs usually consist of an ionizable lipid, a phospholipid, cholesterol, and lipid-anchored PEG,^14^ which are in principle similar to the composition of the lipid membrane of bacteria and enveloped viruses. LNPs are mainly used as delivery agents of RNA or DNA, serving as vaccines or as cancer treatments.^15,16^ Of special interest to the application for disinfection is their sensitivity to surfactants and ethanolic solutions, as well as the possibility of loading the nanoparticles with nucleic acids.

Encapsulating synthetic DNA amplicons within the LNPs allows us to make use of the very sensitive analysis method of quantitative PCR (qPCR), which is able to detect a specific DNA sequence down to a concentration of just one copy per sample.^17^ In the use case as synthetic surrogates, this should allow for the use of very small amounts and low concentrations of LNPs and being still able to detect them.

To evaluate if LNPs encapsulating synthetic DNA can indeed be used as synthetic surrogates for microorganisms, we synthesized such a material and assessed its performance in the validation of surface disinfection procedures.

## Experimental Section

### Formulation of LNPs

LNPs were prepared as previously described.^18^ While a multitude of different formulations of LNPs exist in the literature, these particular LNPs were chosen as no microfluidic chip device is necessary for their formation. The ionizable lipid 306Oi10 was synthesized from 2-isodecyl acrylate (Sigma-Aldrich) and 3,3′-diamino-*N*-methyldipropylamine (Aldrich-Fine Chemicals) in stoichiometric amounts via stirring for 3 days at 90 °C and analyzed by ^1^H and ^13^C NMR.^19^ To prepare the LNPs, DOPE (1,2-dioleoyl-snglycero-3-phosphoethanolamine, Avanti), cholesterol (Sigma-Aldrich), C14-PEG1000 (1,2-dimyristoyl-snglycero-3-phosphoethanolamine-*N*-[methoxy(polyethylene glycol)-1000] (ammonium salt), Avanti), and the previously synthesized 306Oi10 were dissolved in 90% ethanol and 10% 10 nM citrate buffer at molar ratios of 16: 46.5: 2.5: 35*.* The aqueous phase was prepared to comprise an annealed DNA amplicon (62 nt sequence, see the Supporting Information), 10 mM Tris base buffer (pH = 7), and 10 nM citrate buffer, with a final DNA concentration of 1 g/L. Both phases were preheated to 37 °C, and the ethanol phase was added dropwise to the aqueous phase in a 3:1 volumetric ratio and mixed by rapid pipette mixing. The formed LNPs were stabilized with 300 mM NaCl citrate buffer (three times the volume of LNPs) and dialyzed against PBS for 1 h at room temperature using a MWCO 20000 cassette (Gibco).^20^ The DNA blank sample was prepared with the same aqueous phase as described above and pure ethanol as the ethanol phase.

### NMR Data

306Oi10: ^1^H (CDCl_3_, δ): 4.02 (m, 4H), 2.82 (t, 8H), 2.72 (t, 4H), 2.58 (t, 8H), 2.45 (t, 8H), 2.32 (m, 16H), 2.14 (s, 16H), 1.23 (m, 16H), 0.81 (m, 48H)

^13^C (CDCl_3_, δ): 172.90, 64.98, 64.67, 63.11, 62.73, 60.90, 55.99, 51.84, 49.31, 48.28, 45.19, 42.25, 22.84, 22.32, 20.29, 18.03, 14.44, 12.25, 11.46, 11.24.

### Characterization of LNPs

In order to determine the encapsulation rate and to confirm the successful formation of LNPs encapsulating DNA, 10 μL of DNA in LNPs and 10 μL of blank DNA were each diluted with a 190 μL working solution of the Qubit dsDNA HS kit (Invitrogen, Thermo Fisher Scientific), and its fluorescence was measured in the Qubit Fluorometer 3 (Invitrogen, Thermo Fisher Scientific). The encapsulation rate, which is the fraction of DNA encapsulated within the LNPs, is calculated using the following formula
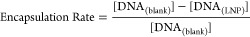
1

The surface potential of the LNPs was measured using a Zetasizer (Malvern). Additionally, the electron microscope Nova NanoSEM 450 (FEI) was used for imaging (acceleration voltage = 10 kV, mag > 20 000x, STEM II detector). For constructing the histogram showing the size distribution, 219 data points were used. NanoDrop 2000c (Thermo Fisher Scientific) was used for recording UV–vis spectra.

### Benzonase Assay

To show that DNA encapsulated in LNPs is protected against enzymatic degradation, LNPs and free DNA were treated with Benzonase (nuclease, purity > 90%, Merck Millipore), purified with the Zymo DNA Clean & Concentrate kit and subjected to electrophoresis on agarose gel (2%, SYBR Gold, Thermo Fisher Scientific) in an E-Gel Power Snap Electrophoresis System (Invitrogen, Thermo Fisher Scientific) for 10 min.

### Surface Test

**Figure 1:**
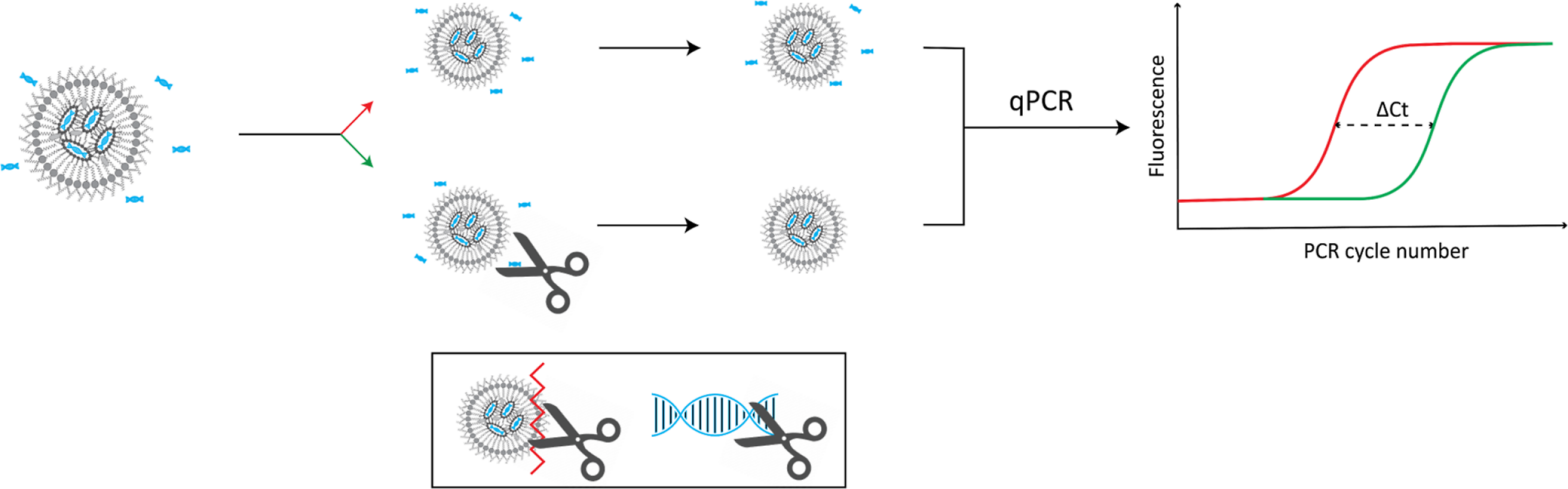
Schematic representation of workflow to determine the amount of intact LNPs in a sample. First, the sample is divided into two portions. One portion is treated with the DNA-degrading enzyme Benzonase (depicted as scissors, DNA in blue), which only degrades free DNA and not DNA that is encapsulated within LNPs (green). The other portion remains untreated (red). By measuring the DNA concentration with qPCR and comparing the DNA concentration in both portions, the amount of encapsulated DNA is determined, which is a direct measure for the amount of intact LNPs.

To investigate the physiochemical properties of the formed LNPs, they were dried on a glass surface, and a surface test was performed. For this, a droplet of 50 μL LNP (5 μg/L) was deposited on a glass surface and air-dried at room temperature. After the droplet was fully dry, the droplet was wiped once with a KimTech wipe (DryWipe, KimTech Science Precision Wipes), premoistened with 100 μL of a test liquid. Following the wiping, the dried droplet was rehydrated with 200 μL of MilliQ water. The liquids used for wiping were ethanol (absolute, VWR chemicals), other disinfectants containing ethanol (advanced hygienic hand sanitising foam (Purell) and Sterillium med and Sterillium gel (both Hartmann)), and a sodium dodecyl sulfate solution in water (SDS, 5 g/L, Sigma-Aldrich). Subsequently, the amount of intact LNPs in the sample was measured as follows: the sample was divided into two portions of equal volume. Part B was treated with the DNAse Benzonase, while part A remained untreated. Both samples were then analyzed via qPCR (LightCycler 480 II, Roche). By comparing the DNA concentration in both samples, the amount of intact LNPs was determined as the free, unencapsulated DNA is expected to degrade, but the encapsulated DNA is expected to remain intact. A schematic representation of the workflow of this analysis is shown in Figure 1. Every data point was measured four times.

## Results and Discussion

### Results

**Figure 2:**
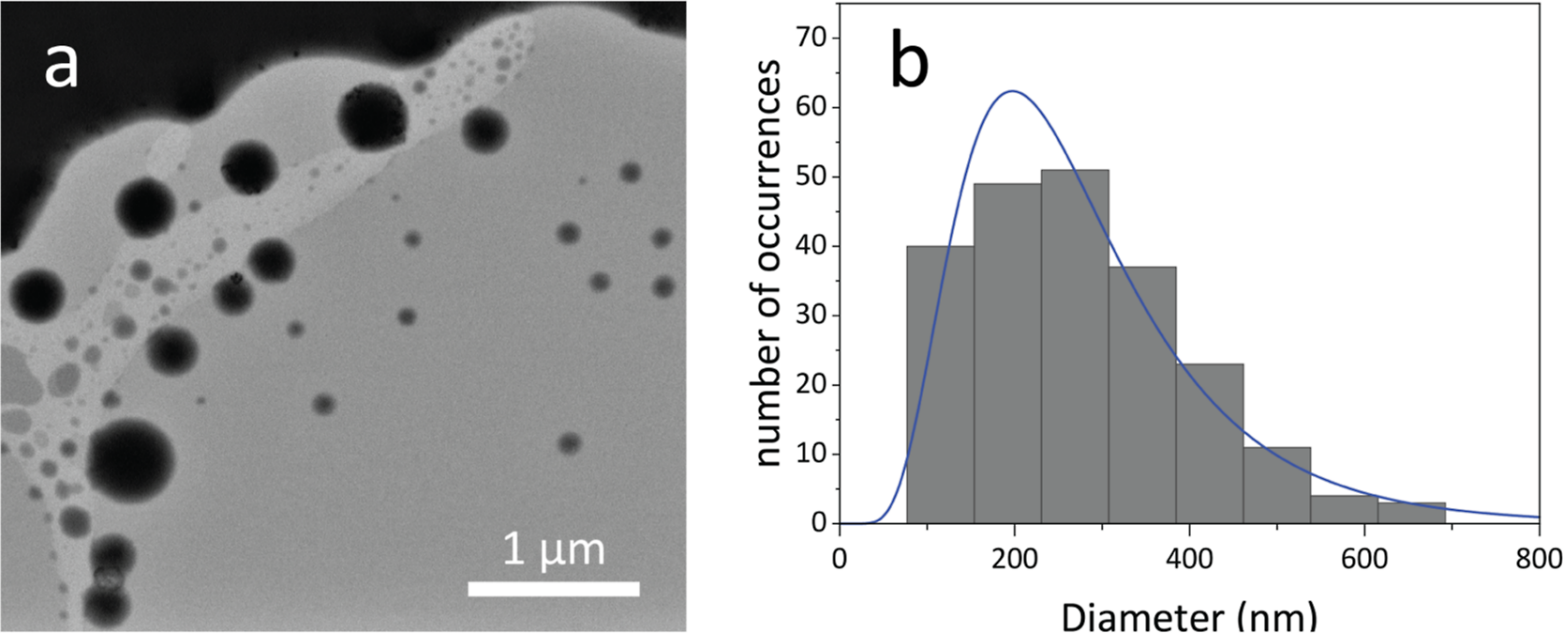
(a) STEM image of LNPs encapsulating DNA depicting the size variety of the particles. The black area in the top left corner is part of the STEM grid. (b) Nanoparticle size distribution histogram obtained from the STEM images following a log-normal distribution (indicated in blue, *n* = 219).

For the preparation of LNPs, we followed a published procedure, which does not require a microfluidic mixing device.^18^ This yielded a white suspension. The encapsulation rate was determined as 90% (by eq 1), and the surface potential of the LNPs was 32 ± 4.5 mV. Measuring the mean diameter via dynamic light scattering was not possible as the LNP size was too polydisperse. This polydispersity stems from the formulation technique of rapid pipette mixing. Using a microfluidic chip device would have led to a more monodisperse size distribution,^21^ but a narrow size distribution is not necessary for the application described here. However, using scanning transmission electron microscopy (STEM), the diameter could be identified as ranging from 150 to 550 nm (see Figure 2).

**Figure 3:**
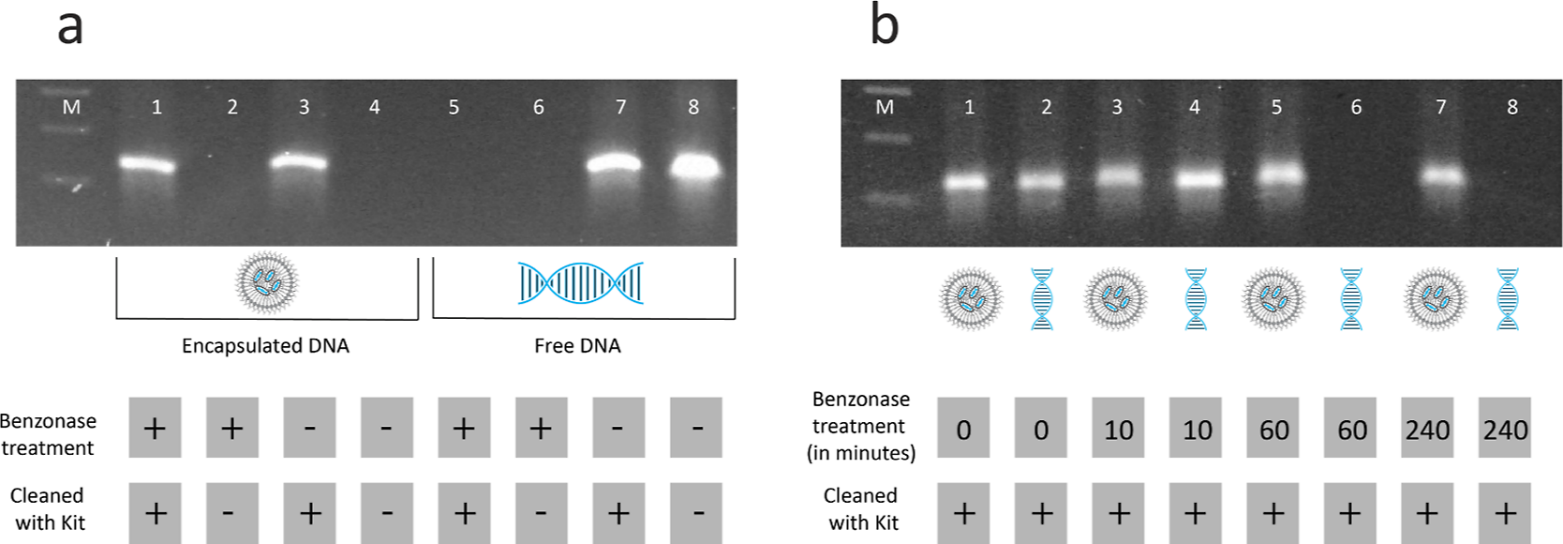
(a) Agarose gel of a DNA amplicon encapsulated in LNPs after treatment with Benzonase and/or a DNA cleanup kit. As a control, the same experiments were performed with free, unencapsulated DNA. (b) Agarose gel of a DNA amplicon encapsulated in LNPs and unencapsulated DNA after treatment with Benzonase and a DNA cleanup kit at different time durations of enzyme incubation.

The successful DNA encapsulation is further visible by agarose gel electrophoresis (Figure 3a): In contrast to free, unencapsulated DNA (lines 7 and 8), the encapsulated DNA does not run on the gel (line 4). Only if the encapsulates are first destroyed by the application of a DNA purification kit involving high concentrations of a chaotropic salt, the DNA is released from the encapsulate making it visible on the gel (line 3). The same analysis can be used to show that the encapsulated DNA is protected from enzymatic decay as encapsulated DNA is still visible after a treatment with Benzonase (line 1), whereas the same Benzonase treatment leads to a full disintegration of unencapsulated DNA (line 5). Furthermore, it is shown that after a time duration of 60 min the unencapsulated DNA is fully degraded (Figure 3b), while encapsulated DNA survives the treatment for more than 240 min.

The above analysis shows that the DNA is only susceptible to enzymatic decay if it is unencapsulated. We can, therefore, use such an analysis to determine whether, in a given sample, DNA is present within an intact lipid particle or whether the particle was destroyed and the DNA was thereby released.

**Figure 4:**
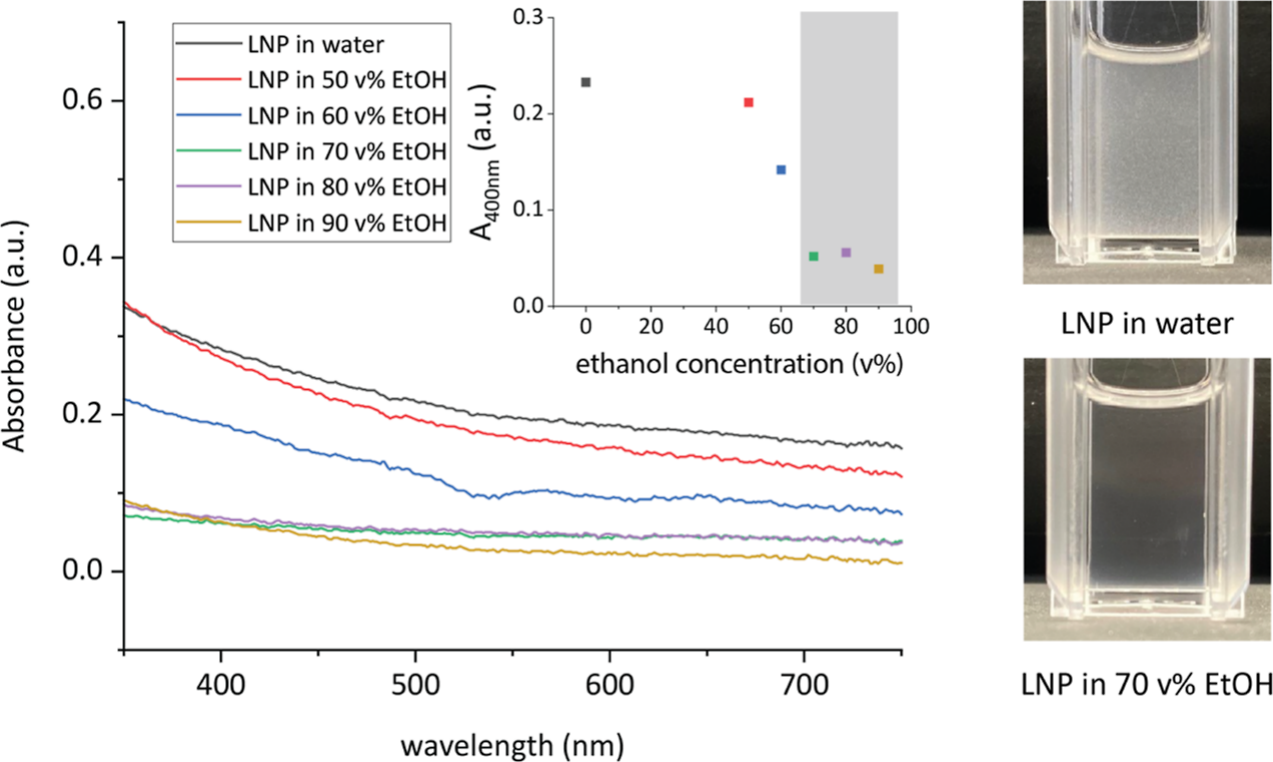
Overlay of UV–vis spectra of the synthesized LNPs (0.35 ng/μL) in aqueous solutions with varying ethanol concentrations. Inset showing the absorbance at 400 nm as a function of the ethanol concentration, where the gray area indicates fully dissolved LNPs. Photographs of 0.35 ng/μL LNPs in aqueous solution and 70 v % EtOH.

For LNPs to be suitable surrogates for bacteria, they need to have the same or similar susceptibility toward disinfection with common disinfectants, which usually comprise high concentrations (≥70 v %) of ethanol.^11^ To investigate the disintegration of LNPs due to contact with ethanol, UV–vis spectra were recorded of LNP dispersions with varying ethanol concentrations, see Figure 4. At low ethanol concentrations, the solutions remained highly turbid, with significant light absorption in the low-wavelength range, consistent with small particle light scattering.^22^ In samples with high ethanol concentrations, the absorbance decreased, and at ethanol concentrations exceeding 70 v % (=56 wt %), the solutions appeared clear and resulted in a significant decrease in optical absorbance, indicating a full dissolution of the LNPs. This behavior of the LNPs corresponds to the one exhibited by bacteria, which are effectively dissolved and the lipid shell is destroyed, when treated with ethanol solutions of concentrations of 70 v % or higher and remain mostly unharmed when ethanol concentrations lower than 50 v % are used for disinfection.^23^

A similar effect can be observed when LNPs are contacted with SDS solutions (see Supporting Information, Figure S1). In this case, it is expected that the surfactant results in a disintegration of the original LNPs and the formation of lipid micelles, very similar to the action of detergents toward microorganisms.^10^

#### Surface Test

The above UV–vis analysis shows that LNPs dissolve in ethanol solutions and in the presence of common detergent solutions (e.g., SDS). However, in order to use LNPs for the validation of disinfection procedures, UV–vis is not a very practical method to determine the intactness of a LNP sample. First, the sensitivity of UV–vis is low (>1 mg/mL concentrations required), and the UV–vis reading of a sample of unknown concentration cannot be used to determine whether the LNPs have been dissolved or whether the LNPs have merely been diluted. These shortcomings can be overcome by using DNA amplicons encapsulated within the LNPs as quantification tools: DNA can be quantified to extreme sensitivities (down to a single-molecule level),^24^ and the enzymatic assay presented further above should be able to assess the integrity of the LNPs within a given sample.

**Figure 5:**
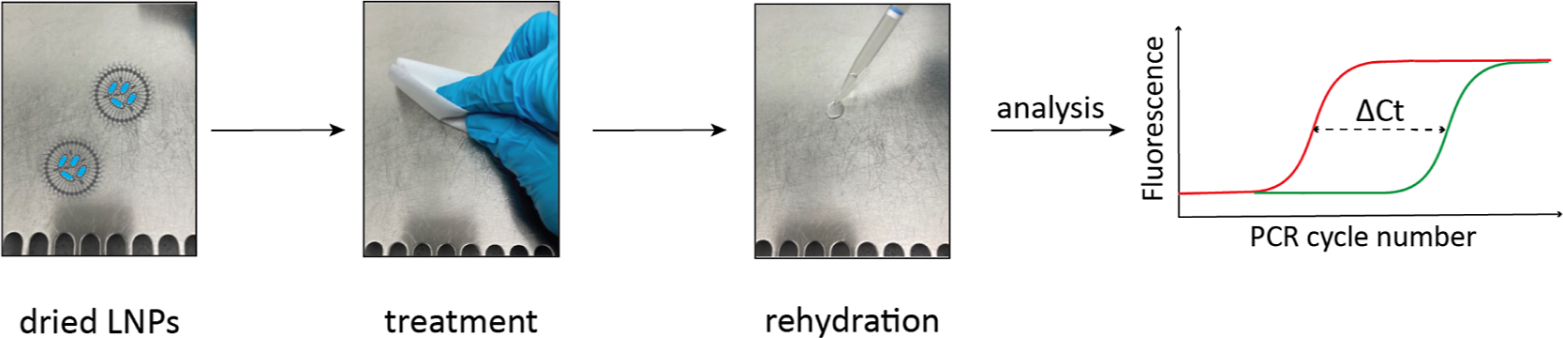
Scheme for the procedure of the surface test. First, LNPs are dried on a surface (here: a steel worktop of a laminar flow bench) and then treated through wiping with a tissue moistened with various liquids. After that, MilliQ water is pipetted on the surface to take the sample (rehydration). Subsequently, the sample is analyzed as described above (Figure 1).

**Figure 6:**
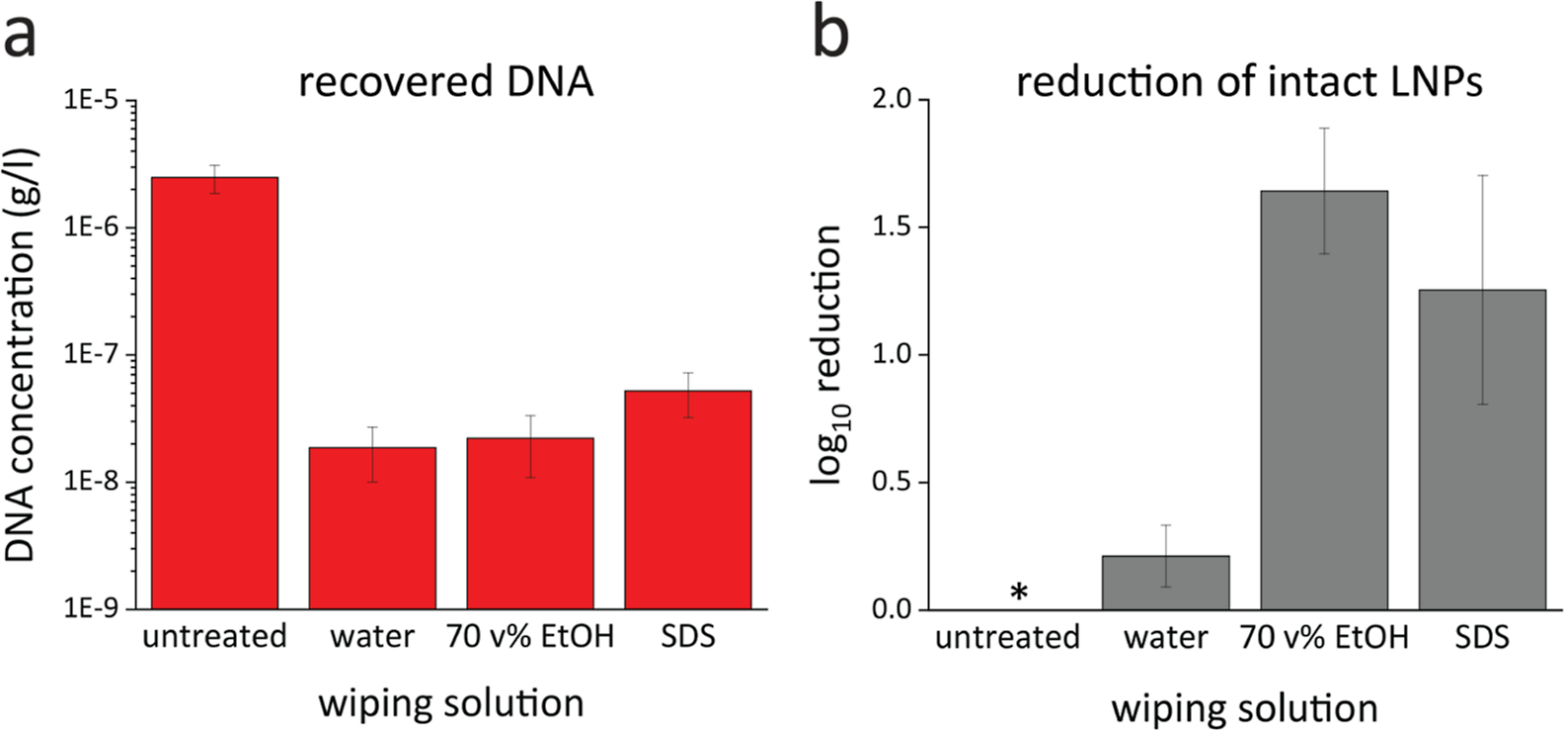
(a) Total DNA recovered from the surface after no wiping or wiping with water, ethanol (70 v %), or SDS. The *y*-axis is logarithmic. A clear dilution effect through wiping is visible and no distinction on which liquid was used for wiping is possible. (b) Reduction of intact LNPs expressed in log_10_ referenced to the untreated sample (*). The log_10_ reduction of intact LNPs is significantly higher when ethanol (70 v %) or SDS is used as a wiping solution compared to wiping with water. Every data point was measured four times.

To test the performance of such a procedure, a surface test was conducted (Figure 5). The as-prepared LNPs carrying a short DNA amplicon were deposited on a glass surface (50 μL/0.005 ng/μL). The LNPs were allowed to dry (2 h), and the surface was subsequently treated through wiping with a tissue moistened with either water, 70 v % ethanol, or an aqueous detergent solution (SDS, 5 g/L). A sample was then taken from the surface by pipetting 200 μL of MilliQ to the surface to rewet and collect the nanoparticles. The total amount of DNA in these samples was measured by qPCR (reading A). Thereby, any diluting (wiping away) of LNPs from the surface can be measured by comparison with the qPCR reading of an untreated sample (no wiping = control reading). Experimental data (Figure 6) show that a comparable amount of LNPs was removed from the surfaces, irrespective of the composition of the wiping solution. In detail, prior to wiping, the qPCR reading was ca. 10 Ct, which was increased to ca. 19 Ct after the wiping. Due to the logarithmic nature of the qPCR reading, the  means that the wiping resulted in a LNP dilution of ca. 500-fold, equivalent to a log_10_ reduction of about 2.7.

In addition, if the sample taken from the surface is first treated by Benzonase and then quantified by qPCR, only the DNA present in intact LNPs is accounted for (reading B) as this DNA is protected by the LNPs from enzymatic decay (see Figure 2). A comparison of this reading B (DNA from intact LNPs) with reading A (total recovered DNA) of a given sample gives information of the intactness of the LNPs after the surface cleaning procedure. As qPCR results in a signal, which is logarithmic to the DNA concentration, the difference of B-A is representative for the fraction of intact LNPs. In Figure 6, these data are reported for the wiping experiments and show that for wiping with water, the fraction of intact LNPs is hardly affected, if compared to a sample with the no wiping step. If, however, the surface is wiped with a cloth dampened with a 70 v % ethanol solution or an SDS-comprising solution, the log_10_ reduction of intact LNPs is ca. 1.5 (see Figure 6). This is equivalent to about >90% of LNPs destroyed by the wiping step. This is in addition to the 500-fold dilution of the LNPs detected by sample A alone.

Consequently, the above procedure allows the quantification of two different parameters from a single sample using only a single measurement device (qPCR cycler): a first reading gives information on whether the surface was physically cleaned/wiped and the second reading performed after an enzymatic decay step gives information on the chemical effectiveness of the disinfectant solution.

**Figure 7:**
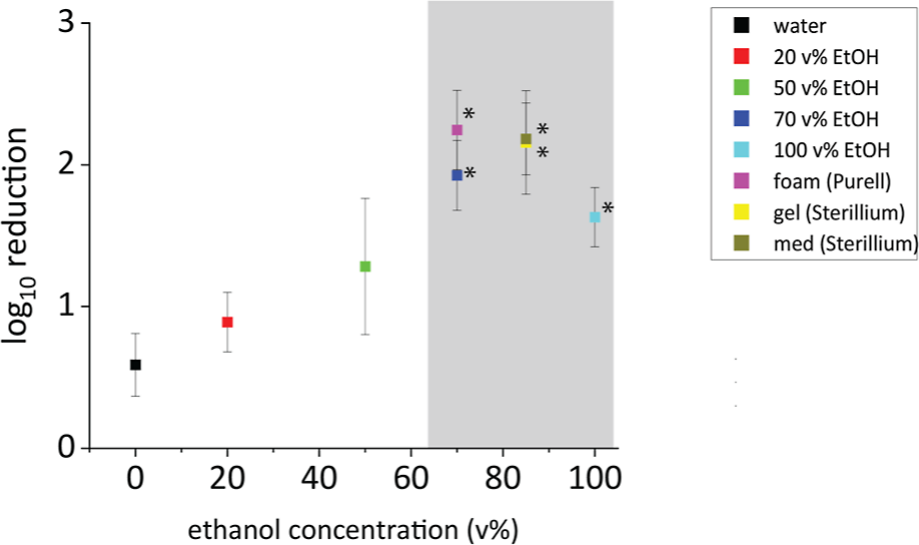
Log_10_ reduction of intact LNPs in relation to the ethanol concentration (v %) of the liquid used for wiping in the surface test. The LNPs are significantly destroyed if an ethanol concentration of ≥70 v % is used, indicated by the gray area. Log_10_ reduction values of ethanol concentrations of ≥70 v % show a significant difference (two sample *t*-test) to water (0 v % ethanol), (*) *p* < 0.05 (*n* = 4). Every data point was measured four times.

As ethanol solutions are such important disinfection solutions, the above procedure was tested with solutions comprising varying ethanol concentrations, as well as commercially available ethanol disinfectants. As shown in Figure 7, low concentrations of ethanol (<50 vol %) did not show a significant difference (p > 0.05, *n* = 4) to wiping with water. Commercial disinfection solutions (Sterillium gel/med and Purell foam) and high concentrations of ethanol (≥70 vol %) resulted in the largest effect and could be clearly distinguished from the pure water control (*p* < 0.05, *n* = 4).

This is in agreement with the improved disinfection performance of these solutions against *Escherichia. coli* and *Serratia marcescens*,^25^ and the log_10_ reduction in CFU of both bacteria after treatment is comparable to the log_10_ reduction of the LNPs studied here, see Table 1.

**Table 1:** Log_10_ Reduction of *S. marcescens,E. coli* (Gram-Negative), *Staphylococcus aureus* (Gram-Positive), Human Immunodeficiency Virus (Enveloped), and Norovirus (Non-Enveloped) after Treatment with Different Disinfectants from the Literature Compared to log_10_ Reduction of Intact LNPs

disinfectants	LNP^a^	S. marcescens^25^	E. coli^25^	S. aureus^25^	HIV^26^	Norovirus^27^
foam (Purell)	4.80	4.7	5.06	4.2		
gel (Sterillium)	4.71	3.12				
70 v % EtOH	4.48	4.7	5.11	4.2	>5.00	3.6

a Dilution and disintegration.

**Figure 8:**
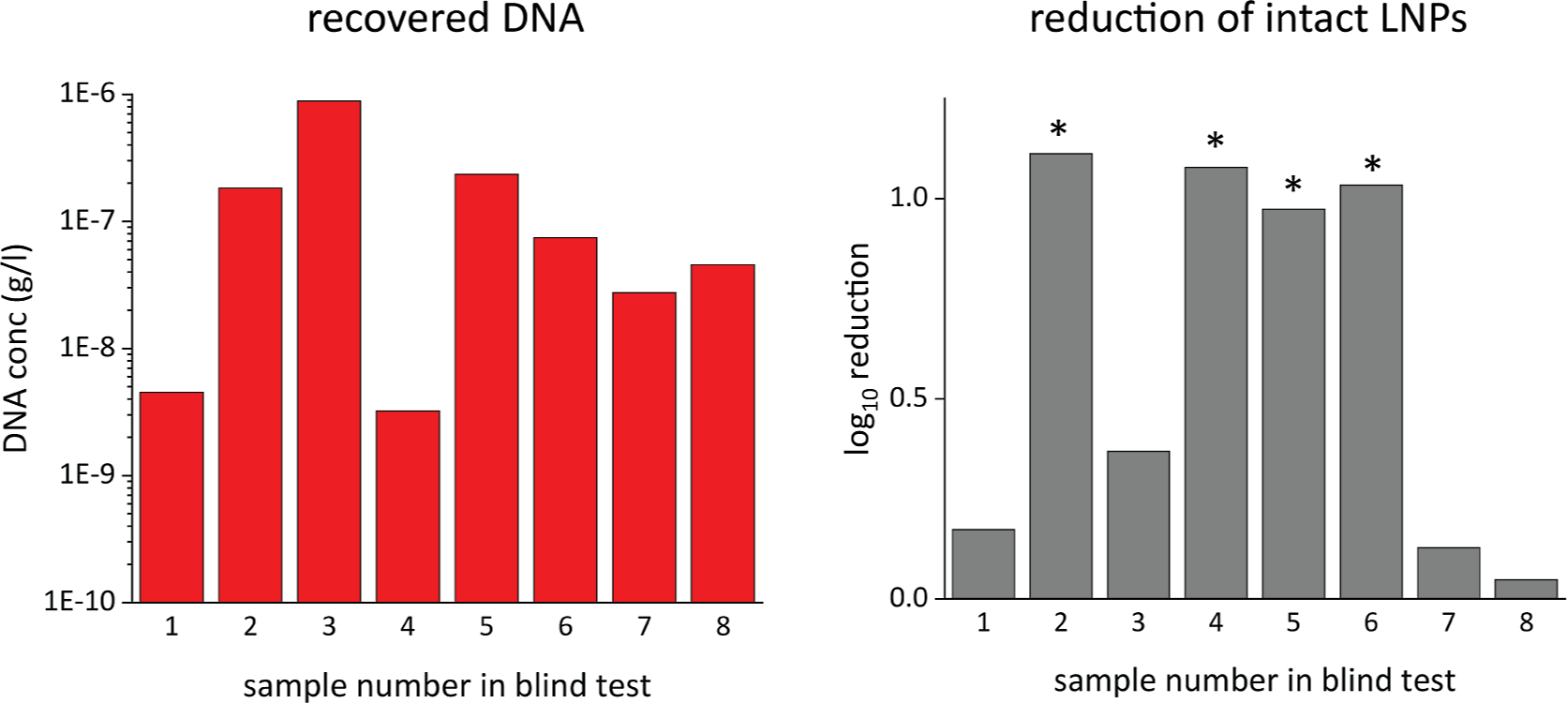
Results of a blind test on glass with sample numbers. Total amount of DNA recovered (left) and associated log_10_ reduction of intact LNPs (right). The asterisk above the bar shows the interpretation that the sample was wiped with 70 v % ethanol. The interpretation was correct for every sample.

In order to show that the procedure described above is indeed useful to determine whether a surface was disinfected with an appropriate disinfectant or merely wiped with water, the DNA amplicon-loaded LNPs were applied to eight different glass surfaces. A second co-worker randomly wiped four of these surfaces with a 70 v % ethanol solution and four with water. In a blind test, the analyst sampled the eight surfaces, determined the log_10_ reduction of intact LNPs for these surfaces, and could thereby correctly identify which surfaces had been treated with the disinfectant and which surfaces had been treated with water (glass: Figure 8, stainless steel: figure S3).

As a control, and to ensure that the readout is indeed a property of the LNPs, and not of the DNA alone, unencapsulated DNA was applied to surfaces and subsequently wiped with various cleaning solutions. It was not possible to determine which liquid was used for wiping (see Supporting Information, Figure S2.) because all samples showed roughly the same log_10_ reduction. This is due to the fact that DNA itself is not degraded by ethanol or SDS, and both chemicals are routinely used in DNA extraction protocols.^28,29^ This further displays the role of the LNPs as DNA encapsulates, which protect the DNA from enzymatic decay but are sensitive to standard disinfectants such as ethanol and detergent solutions.

## Discussion

LNPs treated with ≥70 v % ethanol or treated with SDS show a log_10_ reduction of about 4.5. This reduction includes the dilution through wiping and the disintegration due to ethanol and SDS. Bacteria (Gram-negative and Gram-positive) and viruses (enveloped and non-enveloped) show a log_10_ reduction ranging from 3 to 5 after disinfection with 70 v % ethanol depending on the species, see Table 1. *S. aureus* treated with SDS show a log_10_ reduction in CFU of about 4.^10^ Bacteria, viruses, and LNPs are within the same range of log_10_ reductions when disinfected with ethanol and cleaned with detergents. Therefore, the LNPs are suitable as surrogates for bacteria and viruses in surface disinfection and cleaning validations.

For the application as a surface cleaning validation tool for microbial contamination, LNPs have several advantages over the standard methods.

•UV dyes (e.g., riboflavin) are very straightforward to apply and to detect. However, they can only be used to detect if a surface was physically cleaned, not whether an appropriate chemical was used in the cleaning procedure. In addition, the method is not very suitable for quantitation and cannot detect whether a given dilution factor is achieved during the cleaning procedure.
•Agar growth plates are highly sensitive, can detect single microbial organisms (CFU), and are the most valuable tool to measure the absence of bacterial and fungal contaminations on a surface. However, sample analysis usually requires several days and to quantify the performance of a cleaning procedure the surface must already be contaminated or must be inoculated with a surrogate microorganism prior to cleaning.
•ATP testing combines the rapid readout of the UV dyes with the possibility of indirectly measuring microbial activity. As such, it also requires the presence of microorganisms at concentrations and activities exceeding the detection threshold (4.3 CFU/mL for *E. coli*).^30^ The method suffers from contamination and false positive readings^31^ and is not able to detect the removal of microbial contaminants on a logarithmic scale. Moreover, the method cannot be applied to detect viruses.
•DNA analysis (qPCR) using non-pathogenic viral or phage surrogates has been performed in the past^12^ and offers a surrogate with virtually the same physiochemical properties as the pathogens of interest. To detect viral or phage surrogates, qPCR can be used, but to evaluate disinfection procedures, bacterial cultures are necessary.^32^
•In comparison, LNPs encapsulating DNA amplicons are quite straightforward to synthesize, purify, and store. The method does not involve any microorganism, allows faster analysis than bacterial growth plates, and still results in information on the physical (dilution) and chemical (anti-microbial) effects of a disinfection procedure at very low detection limits (2.5 pg DNA/cm^2^). The different tools for surface cleaning validation are summarized in Table 2.

**Table 2:** Comparison of Surface Cleaning Validation Tools

method	speed	detection limit	measures dilution and disinfection
UV dyes	fast	high	no
agar growth plates	slow	low	yes
ATP testing	fast	moderate	no
DNA analysis (qPCR)	moderate	low	no
LNP encapsulating DNA	moderate	low	yes

Alternative cleaning and disinfection methods which do not involve alcohol or detergents were not tested in this study. However, it may be envisioned that several other disinfection methods could be detected via these surrogates as well. Especially, disinfection methods targeting the chemical integrity of the pathogen membrane or the DNA itself, such as methods relying on reactive oxygen species.

## Conclusions

In this work, we show that LNPs carrying DNA amplicons can be used as surrogates for microorganisms in determining the effectiveness of cleaning procedures. The method allows the detection of the physical (dilution) and chemical (lipid dissolution) action of a cleaning procedure. We anticipate that by varying the size and composition of the LNPs, the behavior and susceptibility toward disinfection agents can be tuned to represent various groups of microorganisms. Also, such DNA-loaded LNPs could be used as models for microorganisms to study their transport via aerosols and via surface-to-surface contact with the goal to understand, combat, and monitor microbial pathogen distribution in manufacturing and health-care settings.

## Supporting Information Available

Additional graphs, UV/vis spectra of LNPs in solutions of different SDS concentrations, surface test on stainless steel with unencapsulated DNA, and list of DNA sequences (PDF)
