# Supplementary material for: Synthetic Microbial Surrogates Consisting of Lipid Nanoparticles Encapsulating DNA for the Validation of Surface Disinfection Procedures
Source: ACS Appl Bio Mater. 2023 Feb 28;6(3):1252–9. doi: 10.1021/acsabm.3c00004 (PMC10031560; doi:10.1021/acsabm.3c00004)
Supplement: Supplementary file 1 — mt3c00004_si_001.pdf [file mt3c00004_si_001.pdf]

Supporting Information

Synthetic microbial surrogates consisting of lipid nanoparticles encapsulating DNA for the validation of surface disinfection procedures

Lara Pfuderer[†], *Wendelin J. Stark*[†] *and Robert N. Grass*[†*]

[†]Institute for Chemical and Bioengineering, ETH Zurich, Vladimir-Prelog-Weg 1, 8093 Zurich, Switzerland.

*Address correspondence to: *rograss@ethz.ch

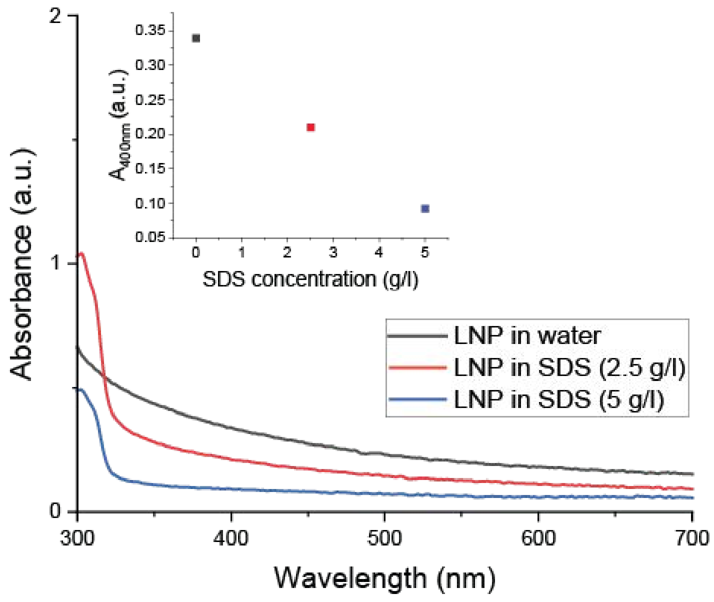

Figure S1. Overlay of UV-Vis spectra of LNP (0.5 ng/µl) in solution with different SDS concentrations. A decline in absorbance with increasing concentration of SDS is visible, meaning that the LNPs dissolve. Inset showing the absorbance at 400nm of LNPs in relation to the concentration of SDS.

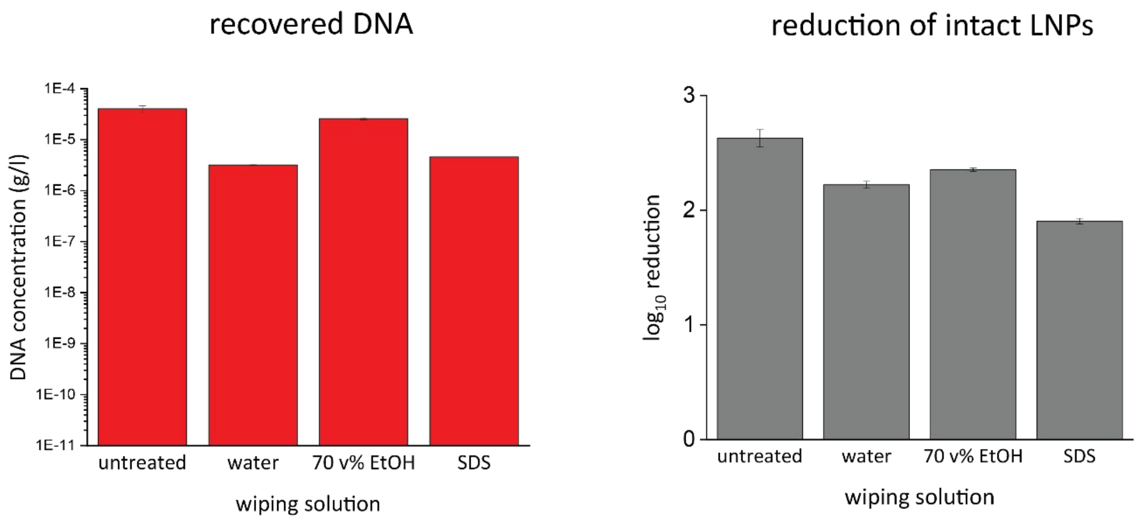

Figure S2. Total recovered DNA and log_{10} reduction after Surface Test with unencapsulated DNA. It is not possible to determine which liquid was used for wiping, because DNA itself is not susceptible towards ethanol or SDS.

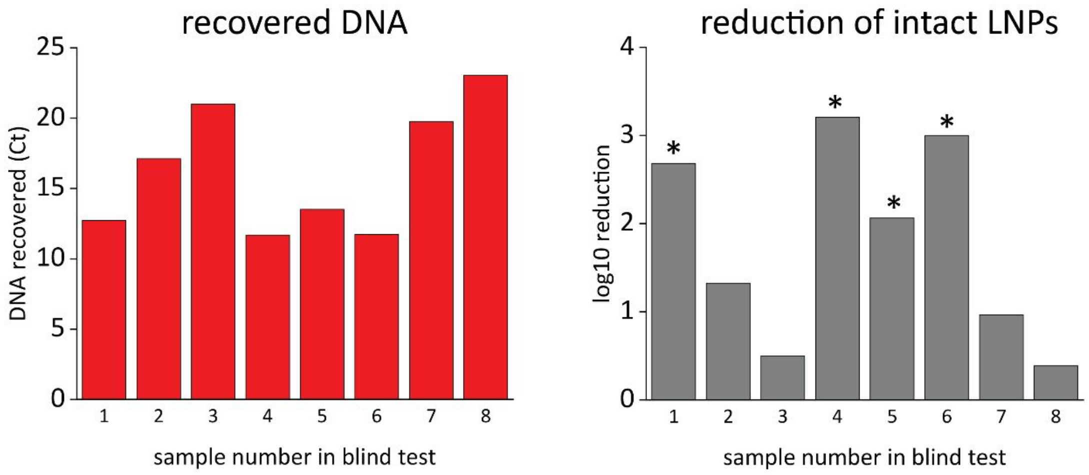

Figure S3. Results of a blind-test on stainless steel with sample number. Amount of total amount of DNA recovered (left) and associated log_{10} reduction of intact LNPs (right). The asterisk above the bar shows the interpretation that the sample was wiped with 70 v% ethanol. The interpretation was correct for every sample.

Table S1. DNA Sequences.

DNA Amplicon	TTC TCT GCC CTT ACG TTT ATC TTA AGG GCC GGT CCA CCA GTT GAA CAC GAA CAA ACC TCT TT
DNA Primer forward	CTC TGC CCT TAC GTT TAT C
DNA Primer reverse	AGA GGT TTG TTC GTG TTC
